# Diagnosis of a Perforated Small Bowel Diverticulum in an Elderly Patient Hindered by a Nuck Cyst: A Case Report

**DOI:** 10.7759/cureus.89660

**Published:** 2025-08-08

**Authors:** Valeria Leal Isla Flores, Cynthia L Nava Palomo, Erick R Guerra Bocanegra, Ana P Rabago Jamaica, Juan H Moreno Juárez

**Affiliations:** 1 Department of General Surgery, Instituto de Seguridad y Servicios Sociales de los Trabajadores del Estado (ISSSTE) Clínica Hospital Constitución, Monterrey, MEX

**Keywords:** elderly patients, nonspecific abdominal pain, nuck cyst, small bowel diverticulitis, surgical acute abdomen

## Abstract

Small intestinal diverticulosis is a rare condition, often asymptomatic until complicated with diverticulitis, bleeding, obstruction, or perforation. It predominantly affects elderly men and may present concomitantly with colon diverticulosis. We report the case of a 94-year-old Hispanic woman brought to the emergency department presenting with an acute abdomen. On physical examination, a tense, tender mass was seen in her left inguinal region, initially suspected as an incarcerated inguinal hernia. An abdominal computed tomography scan showed that this mass corresponded to a Nuck cyst. An encapsulated abscess adjacent to a jejunal segment and pneumoperitoneum were also seen, prompting an emergency surgical intervention. Intraoperative findings included a perforated diverticulum in the distal jejunum, as well as extensive diverticulosis involving the ileum and left colon. Resection and anastomosis were performed. Small bowel diverticulitis remains challenging to diagnose preoperatively, and more so in an elderly patient. This case report highlights the role of imaging in discerning among the differential diagnosis for acute abdomen in the elderly patients. It also demonstrates how rare pathologies, such as the Nuck cyst, could masquerade a different etiology and hinder its diagnosis.

## Introduction

Small intestine diverticulosis was first described by Sömmering in 1794 [1-6]. The reported incidence ranges from 0.3% to 2.3% annually, with autopsy prevalence estimates between 0.02% and 8% [2,5,7-11]. The proposed etiology involves weakness of the intestinal wall at the penetration site of the vasa recta near the mesenteric border, combined with intestinal dyskinesia caused by dysfunction of the smooth muscle or the myenteric plexus (also known as the Auerbach's plexus, part of the enteric nervous system), leading to segmental high intraluminal pressures [1,5,6,8-10,12]. This facilitates mucosal and submucosal protrusion through the muscular layer, resulting in a false diverticulum (pseudodiverticulum, composed only of mucosa and submucosa) formation [1,3,4,7]. The sex distribution is approximately 2:1 men to women, with the onset typically between the seventh and eighth decades of life [1,2,4,6,8,10]. Many patients remain asymptomatic. However, up to 29% may present nonspecific symptoms such as chronic abdominal pain, meteorism, early satiety, diarrhea, or steatorrhea [1-3,5,6,12]. Others can show symptoms of acute abdomen associated with complications, including diverticulitis, bleeding, obstruction, or perforation [1,6,9,10,12]. In case of perforation, its mortality can increase up to 40% [3]. The following case report describes a woman in her 10th decade of life presenting with acute abdominal pain secondary to a perforated jejunal diverticulum whose diagnosis was hindered by another rare condition.

## Case presentation

A 94-year-old Hispanic woman without significant chronic degenerative conditions, with previous surgical history of conventional complicated appendectomy requiring a right hemicolectomy, right total hip replacement, and phacoemulsification with bilateral intraocular lens placement, is brought to the emergency department by a family member. Her symptom onset started two days before admission, characterized by localized abdominal pain in the left and right iliac fossa, rated 8 out of 10 on the visual analog scale. The pain was sharp, radiating diffusely across the abdomen, accompanied by nausea and two episodes of vomiting of gastric content. Her last bowel movement was 24 hours before admission, with no reported abnormalities.

On physical examination, her vital signs were stable, and she was disoriented in the time sphere. Her abdomen was distended with active peristalsis, tympanic on percussion, and tender in all four quadrants, with exacerbation of pain to palpation in the left iliac fossa. A palpable 9 x 6 cm (3.5 x 2.36 inch) mass was noted over her left inguinal ligament, with normal skin tone, tender and tense to palpation, nonreducible, and not modified by the Valsalva maneuver.

**Figure 1:**
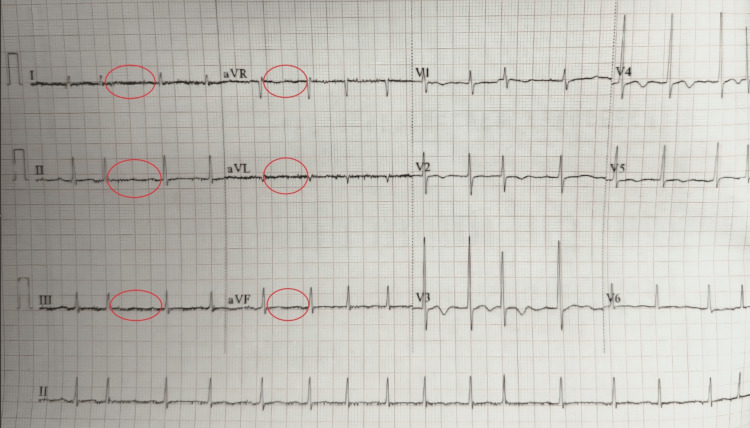
EKG showing atrial fibrillation (circled in red) with rapid ventricular response (HR 102 bpm) and T inversion not previously diagnosed EKG: electrocardiogram; HR: heart rate; aVL: augmented vector left; aVR: augmented vector right; aVF: augmented vector foot

Laboratory results revealed leukocytosis (25.5 x 10^3^/μL), elevated C-reactive protein (CRP; 186 mg/L) (Table 1), abnormal urinalysis (Table 2), and an electrocardiogram showing atrial fibrillation with rapid ventricular response (congestive heart failure, hypertension, age, diabetes mellitus, stroke, vascular disease, age, sex score of 3) (Figure 1). Initial conservative management was undertaken with the suspicion of incarcerated inguinal hernia, starting with IV fluids, IV analgesia, and antibiotics (1 g ceftriaxone BID and 500 mg metronidazole TID).

**Table 1:** Bloodwork results CRP: C-reactive protein

Bloodwork	Result	Reference value
Leucocytes	25.5 × 10³/µL	4.4-11.30 × 10³/µL
Neutrophils	95.60%	41-73%
Hemoglobin	11 g/dL	12.30-15.30 g/dL
Hematocrit	31.80%	36-48%
Platelets	337 × 10³/µL	150-400 × 10³/µL
Na	140.8 mmol/L	136-145 mmol/L
K	3.5 mmol/L	3.5-5.1 mmol/L
Cl	108 mmol/L	98-107 mmol/L
CRP	186 mg/dL	0-3 mg/dL

**Table 2:** Results of urianalysis

Urinalysis	Result	Reference value
Color	Brown	Yellow
Density	1.026	1.015-1.025
pH	6	5-7
Leukocytosis	1+ leu/µL	Negative
Nitrites	Positive	Negative
Proteins	1+ mg/dL	Negative
Glucose	Negative	Negative
Blood	1+ mg/dL	-
Erythrocytes	2-3/field	0-5/field
Leucocytes	8-10/field	0-5/field

**Figure 2:**
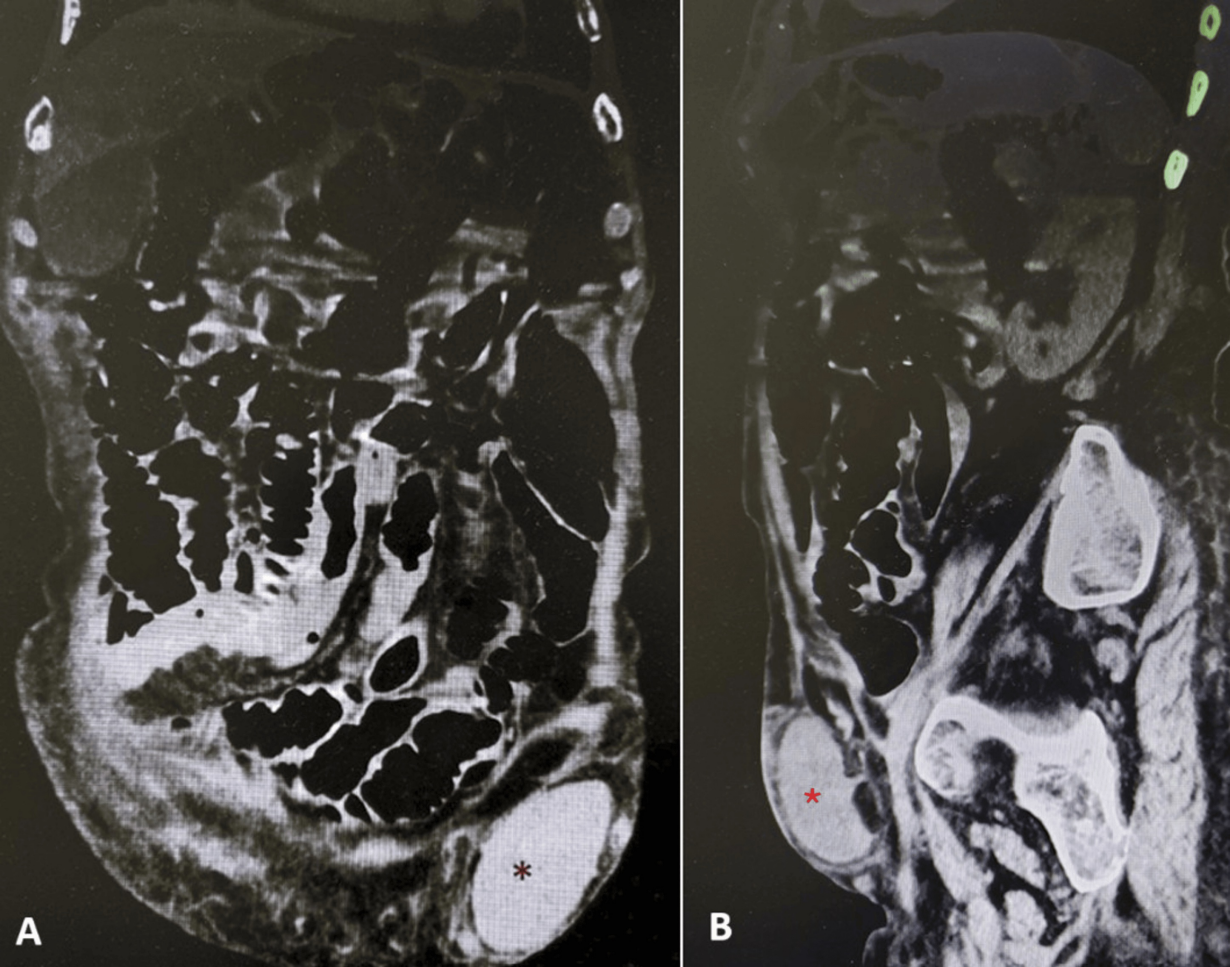
CT coronal (A) and sagittal (B) projections, showing a cystic lesion in the left inguinal canal with no apparent communication with the abdominal cavity (dark red and bright red asterisks) CT: computed tomography

**Figure 3:**
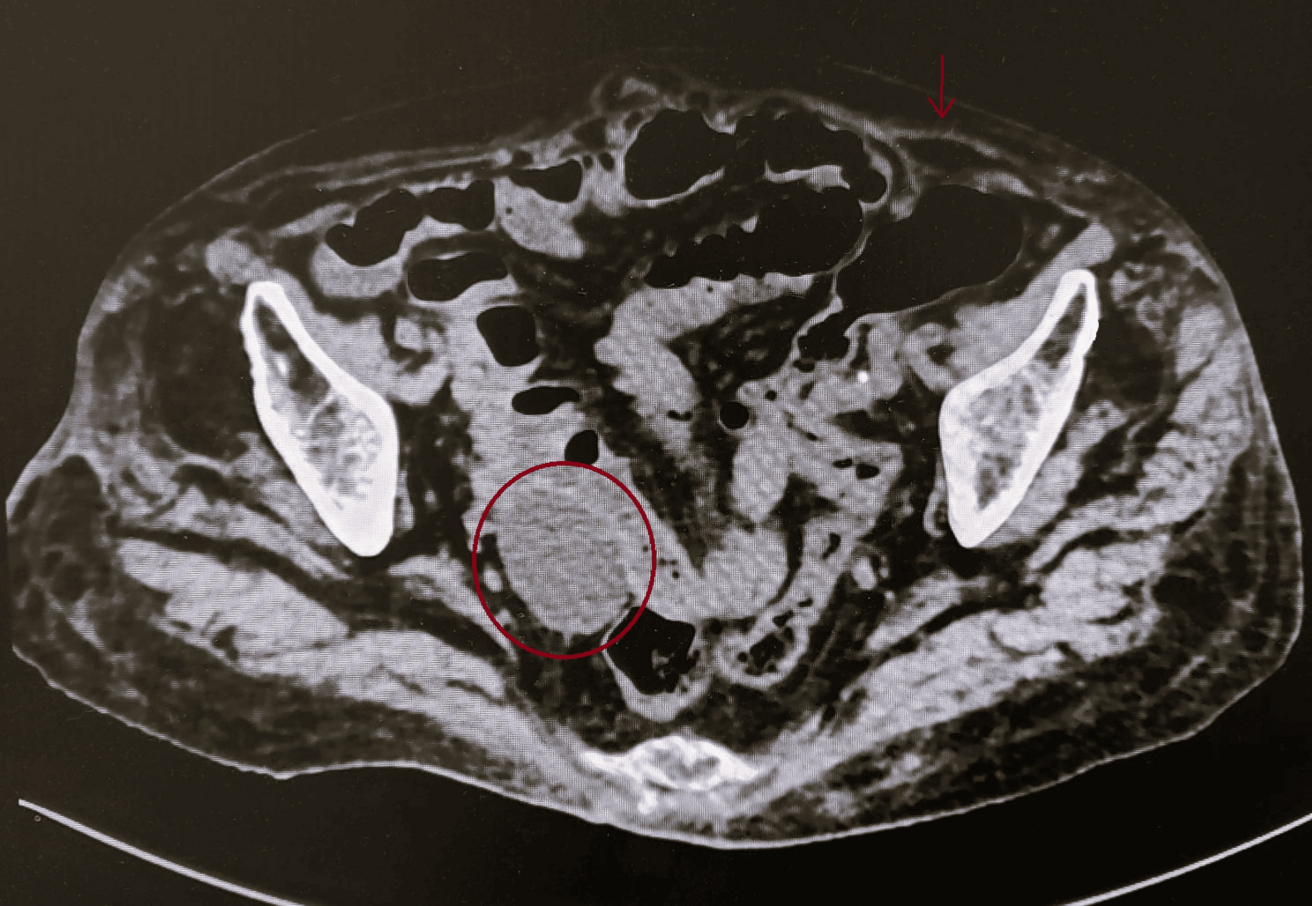
CT axial projection showing an abscess contained adjacent to a jejunal loop (red circle) and pneumoperitoneum (red arrow) CT: computed tomography

A contrast-enhanced computed tomography (CT) scan revealed a cystic lesion along the left inguinal ligament, without communication to the abdominal cavity, as well as an abscess adjacent to a small intestinal loop and free air (Figures 2, 3), prompting emergency surgical intervention.

**Figure 4:**
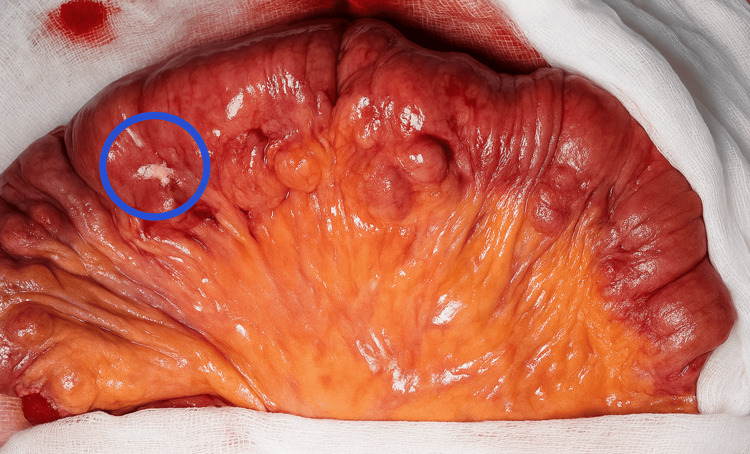
Jejunal loop with the diverticular perforation identified by a blue circle. Notice the large base diverticula along the mesenteric border

**Figure 5:**
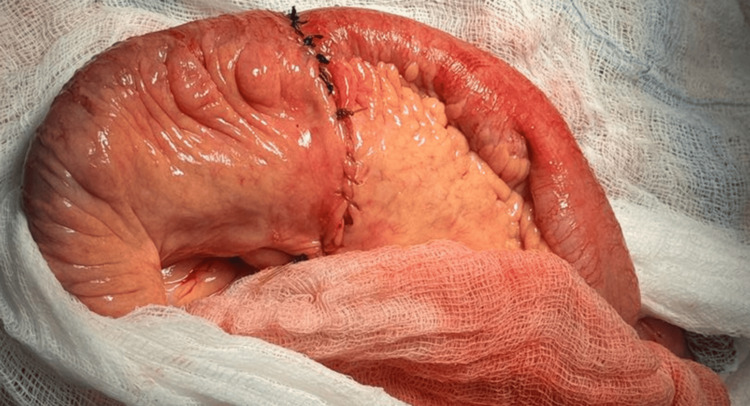
Terminal jejunojejunal double-layer manual anastomosis using polyglycolic acid and silk. Closure of the mesenteric breach with chromic gut suture is visible

Exploratory laparotomy confirmed the presence of a cystic lesion on the left inguinal ligament, without intra-abdominal communication. Scarce inflammatory fluid was obtained when opening the abdominal cavity through laparotomy, and an extensive diverticulosis was observed involving the totality of the jejunum, ileum, and left (remaining) colon. Approximately 50 cm (17.7 inches) from the Treitz ligament, an abscess contained by a mesenteric patch and surrounding bowel loops was evidenced. The surrounding bowel loops were freed; the abscess was drained, and a perforated jejunal diverticulum was identified (Figure 4). A segmental resection of 20 cm (7.9 inches) was performed with a primary end-to-end jejunojejunal manual anastomosis (Figure 5).

The initial postoperative course was favorable, and the patient tolerated oral intake by the second day. Thromboprophylaxis (2.5 mg of apixaban BID) started 48 hours after the surgery. Her antibiotic regimen, selected before the surgery, was continued in the postoperative period. Although they are not the preferred antibiotic agents for diverticulitis (ciprofloxacin and metronidazole chosen as the gold standard), due to the lack of availability in our public hospital, we opted to continue with ceftriaxone and metronidazole. Nonetheless, she died on the fourth postoperative day due to nosocomial pneumonia and cardiovascular complications. In brief, we present Table 3 with the key clinical and surgical data exhibited in this case.

**Table 3:** Summary table CRP: C-reactive protein

Steps	Description
Presenting symptoms	About 48 hours of intense abdominal pain, radiating diffusely throughout the abdomen, accompanied by nausea and two episodes of vomiting
Physical exploration	Vital signs were stable. Distended abdomen with peristalsis, tender in all four quadrants, pain to palpation in the left iliac fossa. A palpable mass on her left inguinal region, tense to palpation, nonreducible, and not modified by the Valsalva maneuver
Laboratory	Leukocytosis (25.5 × 10^3^/μL), elevated CRP (186 mg/L), abnormal urinalysis
Imaging	Cystic lesion along the left inguinal ligament, without communication to the abdominal cavity, abscess adjacent to a small intestinal loop, and free air
Surgical findings	Cystic lesion on the left inguinal ligament, no communication to the abdominal cavity. Inflammatory fluid, diverticulosis in the small and large intestines, contained abscess, and perforation of the diverticulum at the distal jejunum
Surgical intervention	Laparotomy, abscess drainage, resection, primary end-to-end jejunojejunal manual anastomosis

## Discussion

Intestinal perforation as the presentation of small bowel diverticulitis is a rare entity, with reported incidence ranging from 2.1% to 7% [4,7]. Santos Rancaño et al. even report a lower incidence of 0.3%-1%. It may be suspected in patients with a history of colonic diverticular disease, as both conditions can present concomitantly in up to 75%-80% of cases [3,10]. In this patient, she presented an extensive diverticular disease, involving her remaining left colon (because she underwent a right hemicolectomy) and the totality of her small intestine. Colonic diverticulosis is far more common and often diagnosed incidentally on colonoscopy, with an estimated prevalence in Western societies of 5% for patients under the age of 40 years and a prevalence of 50% in patients above the age of 60 years [13]. The suspected pathophysiology of both small bowel and colonic diverticulosis is thought to be the same. Literature shows that small bowel diverticulosis primarily develops in the proximal jejunum (75%), due to the larger caliber of the vasa recta, which favors the protrusion of the mucosal and submucosal layers [11]. In the distal jejunum, they occur in approximately 20% of cases and in the ileum in about 5% [3,8,11]. They are typically multiple and can vary in size from a few millimeters up to more than 26 cm [8,12]. Because these are technically pseudodiverticula, lacking muscularis propria, their walls are thin and fragile, predisposing them to perforation [4,5].

Small intestinal diverticulitis remains clinically silent until it becomes complicated, or it can remain undiagnosed when presenting with nonspecific symptoms, often requiring exclusion of more common pathologies [10]. Complications are observed in only about 10% of cases, with perforation being the most frequent (2%-6%) [1,3,10,14]. Laboratory markers that guide the diagnosis of a diverticular perforation are leukocytosis above 10 x 10^3^/μL and a CRP value over 150 mg/L. Manifestations include localized abscesses or generalized peritonitis [2]. The mortality rate for jejunal diverticulitis ranges from 0% to 5%, but perforation can increase mortality up to 40% [3-5,8,11]. Surgical intervention for perforated small bowel diverticulitis is indicated in unresponsive patients to conservative management or without the availability of interventional radiology; resection and primary anastomosis is the indicated treatment.

CT imaging remains the modality of choice for diagnosing complications of small bowel diverticulitis, although in many cases, definitive diagnosis is made intraoperatively [5,11,14,15]. The characteristic CT findings include mural thickening, fat stranding, abscess, and pneumoperitoneum [4,8,10,11]. In Figure 1, an abscess adjacent to the small intestine measuring approximately 6 cm (2.36 inches) in its greatest axis is observed, marked with a red circle, along with free air, indicated by the red arrow. Figure 2 shows the Nuck cyst, indicated with an asterisk.

This case presented an uncommon clinical distractor. She showed signs of acute abdomen, with initial suspicion of an incarcerated inguinal hernia. Because she was stable, conservative management was started, and a CT scan was requested. The images revealed a Nuck cyst, which is a rare pathology often mistaken for an inguinal hernia, which can sometimes cause pain [2,16,17]. The Nuck cyst was first described by Anton Nuck in 1691, and the first published case was seen in 1892 by Coley [2,16,17]. It is also referred to as a female hydrocele of the Nuck canal, thought to be caused by an incomplete closure of the processus vaginalis during embryogenesis, generating an accumulation of liquid and the formation of a cyst [2,16,17]. Its estimated incidence is only reported in the pediatric population, reaching up to 1% between the ages of 1 and 14 years old [2]. Although it is a congenital pathology, it is not always diagnosed promptly because it may be asymptomatic, may be confused with other pathologies, may be misdiagnosed, and its size and location along the inguinal canal and labia may vary with time [2,16,17]. Surgical management can be offered, but there are no guidelines to define adequate surgical treatment [2]. The patient was not able to give more information on how long she had noticed a mass in her inguinal area or if she had noted changes in its position or dimensions. Her family members were unaware of the presence of a mass in her inguinal area.

In our case, the Nuck cyst was left in place, and we concentrated on solving the abdominal emergency. The exploratory laparotomy revealed extensive diverticular disease involving the jejunum, ileum, and left colon. Figure 3 illustrates the patient’s jejunal loop with multiple diverticula ranging from 1 to 3 cm in diameter, located along the mesenteric border, as well as the perforation site marked with a blue circle. A segmental resection with primary anastomosis was performed, which is the preferred surgical approach, involving resection of only the affected segment to prevent short bowel syndrome [3-5,7,8,10,12,15]. It was challenging to find the optimal site for resection due to the multiple diverticulosis present proximally and distally to the perforation, as we wanted to evade having a diverticulum in the anastomosis line. If surgical expertise and resources are available, the same procedure could be performed laparoscopically to benefit from minimally invasive surgery.

## Conclusions

Small intestinal diverticulosis is a rare condition, often asymptomatic until complicated with diverticulitis, bleeding, obstruction or perforation. It remains difficult to diagnose it preoperatively and more so in an elderly patient. CT imaging is the diagnostic modality of choice and surgical resection remains the definitive treatment for complicated diverticulitis. This case underscores the importance of considering small intestinal diverticulitis as a cause of acute abdomen in the elderly patients, and it highlights the role of imaging in discerning among its differential diagnosis, even rare pathologies that can delay its surgical management, like the Nuck cyst present in this patient.
